# A rational roadmap for SARS‐CoV‐2/COVID‐19 pharmacotherapeutic research and development: IUPHAR Review 29

**DOI:** 10.1111/bph.15094

**Published:** 2020-07-19

**Authors:** Steve P.H. Alexander, Jane F. Armstrong, Anthony P. Davenport, Jamie A. Davies, Elena Faccenda, Simon D. Harding, Francesca Levi‐Schaffer, Janet J. Maguire, Adam J. Pawson, Christopher Southan, Michael Spedding

**Affiliations:** ^1^ Chair, Nomenclature and Standards Committee of the International Union of Basic and Clinical Pharmacology (NC‐IUPHAR), School of Life Sciences University of Nottingham Nottingham UK; ^2^ Curator, Guide to PHARMACOLOGY (GtoPdb), Deanery of Biomedical Sciences University of Edinburgh Edinburgh UK; ^3^ Executive Committee, NC‐IUPHAR University of Cambridge Cambridge UK; ^4^ Principal Investigator, Guide to PHARMACOLOGY (GtoPdb), Executive Committee, NC‐IUPHAR, Deanery of Biomedical Sciences University of Edinburgh Edinburgh UK; ^5^ Database Developer, Guide to PHARMACOLOGY (GtoPdb), Deanery of Biomedical Sciences University of Edinburgh Edinburgh UK; ^6^ First Vice‐President and Chair of Immunopharmacology Section, International Union of Basic and Clinical Pharmacology (IUPHAR) Hebrew University of Jerusalem Jerusalem Israel; ^7^ University of Cambridge Cambridge UK; ^8^ Senior Curator, Guide to PHARMACOLOGY (GtoPdb), Executive Committee, NC‐IUPHAR, Deanery of Biomedical Sciences University of Edinburgh Edinburgh UK; ^9^ Deanery of Biomedical Sciences University of Edinburgh Edinburgh UK; ^10^ TW2Informatics Ltd Gothenburg Sweden; ^11^ Secretary‐General, International Union of Basic and Clinical Pharmacology (IUPHAR) and Spedding Research Solutions SAS Le Vesinet France

## Abstract

In this review, we identify opportunities for drug discovery in the treatment of COVID‐19 and, in so doing, provide a rational roadmap whereby pharmacology and pharmacologists can mitigate against the global pandemic. We assess the scope for targeting key host and viral targets in the mid‐term, by first screening these targets against drugs already licensed, an agenda for drug repurposing, which should allow rapid translation to clinical trials. A simultaneous, multi‐pronged approach using conventional drug discovery methods aimed at discovering novel chemical and biological means of targeting a short list of host and viral entities which should extend the arsenal of anti‐SARS‐CoV‐2 agents. This longer term strategy would provide a deeper pool of drug choices for future‐proofing against acquired drug resistance. Second, there will be further viral threats, which will inevitably evade existing vaccines. This will require a coherent therapeutic strategy which pharmacology and pharmacologists are best placed to provide.

**LINKED ARTICLES:**

This article is part of a themed issue on The Pharmacology of COVID‐19. To view the other articles in this section visit http://onlinelibrary.wiley.com/doi/10.1111/bph.v177.21/issuetoc

**Abbreviations**
3CL_pro_
: 3C‐like proteinase of the virus
ADRP: ADP‐ribose‐1″‐phosphatase
ARDS: acute respiratory distress syndrome
BPS: British Pharmacological Society
CARD: caspase activation and recruitment domain
CoV: coronavirus
E: envelope protein of the virus
FRET: Förster Resonance Energy Transfer
GtoPdb: BPS/IUPHAR Guide to PHARMACOLOGY database
IUPHAR: International Union of Basic and Clinical Pharmacology
M: membrane glycoprotein of the virus
MERS: Middle East respiratory syndrome
N: nucleocapsid protein of the virus
nsp: non‐structural protein of the virus
PAMP: pathogen‐associated molecular pattern
PL_pro_
: papain‐like proteinase of the virus
RBD: receptor‐binding domain
S: spike glycoprotein of the virus
SADS: swine acute diarrhoea syndrome
SARS: severe acute respiratory syndrome
TM: transmembrane

## 1 INTRODUCTION

PubMed has already accumulated a vast repository of information on SARS‐CoV‐2/COVID‐19, which increases on a daily basis (on 2020‐03‐23, there were 1,369 hits for COVID‐19; this number more than doubled in the space of 2 weeks, so that by 2020‐04‐06 there were 2,780 hits in PubMed for COVID‐19). Clearly, there is a need to summarize this information critically and prioritize the elements, which are constructive and useful for each individual sector. This review suggests priorities for how drug discovery and development might be rationally focussed for the rapid identification and successful translation of therapeutic agents to treat COVID‐19.

Given the urgency of the current situation, clearly, initial drug discovery should focus on repurposing licensed drugs, as dosage and safety information are largely to hand. Unfortunately, there is controversy over proof of efficacy for essentially all the potential repurposed agents for which preliminary and, in many cases, non‐peer reviewed data have surfaced. Some of this controversy is addressed below, but efforts are underway from both WHO and NIH to coordinate larger, higher powered and better controlled studies in an attempt to demonstrate efficacy unequivocally. As a “second wave,” *de novo* discovery focussing on novel agents may allow future refinement and capacity to treat patients who are unable to be treated by, or are unresponsive to, the repurposed agents, but it would be very unlikely to have these new drugs available to treat the current crisis.

The IUPHAR/BPS Guide to PHARMACOLOGY (GtoPdb) is an open‐access database, developed by the International Union of Basic and Clinical Pharmacology (IUPHAR) and the British Pharmacological Society (BPS). It provides expert‐curated descriptions of almost 3,000 human proteins and over 10,000 ligands, including more than 1,400 approved drugs. Management of the resource is the responsibility of the Nomenclature and Standards Committee of IUPHAR (NC‐IUPHAR), which acts as the scientific advisory and editorial board. The committee has an international network of over 700 expert volunteers organized into ∼60 subcommittees dealing with individual target families. The database is notably enhanced through the continued linking of relevant pharmacology with key immunological data types as part of the IUPHAR Guide to IMMUNOPHARMACOLOGY (supported by the Wellcome Trust) and by a major new extension, the IUPHAR/MMV Guide to Malaria PHARMACOLOGY (in partnership with the Medicines for Malaria Venture). The GtoPdb team centred at the University of Edinburgh have constructed a resource (Coronavirus Information Portal), which provides a precis of the current understanding about the virus and potential associated drug targets and drugs. As with the other databases, the emphasis of the curation process is on stringent provenancing of the information provided, although inevitably the current situation limits the capacity for triangulation of data.

## 2 THE VIRAL CYCLE AND VIRALLY ENCODED POTENTIAL DRUG TARGETS

For general reviews of the coronaviruses, see Masters (2006); Fehr and Perlman (2015); de Wit, van Doremalen, Falzarano, and Munster (2016); Zumla, Chan, Azhar, Hui, and Yuen (2016); Cui, Li, and Shi (2019); Desforges et al. (2019); and Song et al. (2019). SARS‐CoV‐2 is a betacoronavirus, a lipid‐enveloped, single‐stranded and positive sense RNA virus. Other human coronaviruses include alphacoronaviruses, such as human coronavirus‐229E (HCoV‐229E), and betacoronaviruses, such as SARS‐CoV and MERS‐CoV (responsible for the Middle East respiratory syndrome) (for review, see Zumla et al., 2016; Corman, Muth, Niemeyer, & Drosten, 2018; Pillaiyar, Meenakshisundaram, & Manickam, 2020). More than 200 viral types have been associated with the common cold, of which 50% of infections are rhinovirus, but also include respiratory syncytial virus, influenza and coronaviruses, particularly HCoV‐229E. Although HCoV‐229E is regarded as “relatively benign” since monocytes are much more resistant to infection, it does rapidly kill dendritic cells (Mesel‐Lemoine et al., 2012).

**FIGURE 1:**
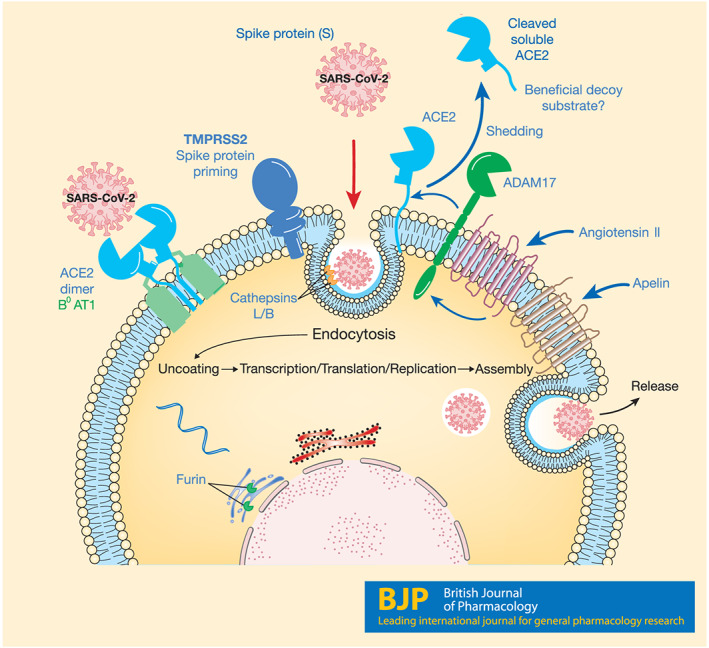
The SARS‐CoV‐2 life cycle. The novel virus uses ACE2 to attach to target cells, including epithelial and endothelial cells, particularly in the lungs. SARS‐CoV‐2 requires the camostat‐sensitive serine proteinase TMPRSS2 to prime the spike protein for fusion and internalization. Thereafter, host cellular processes are exploited for viral replication and release from the cell. ACE2 is also expressed in high levels in the GI tract, where it is associated with B^0^AT1/SLC6A19 that actively transports neutral amino acids across the apical membrane of epithelial cells. The serine proteinase ADAM17, present on cell surfaces, cleaves ACE2 to release an ectodomain of ACE2, including the active site, into the circulation. This circulating form of ACE2 may also bind SARS‐CoV‐2, but this complex is predicted not to internalize and therefore could be exploited as a beneficial viral decoy. Recombinant ACE2 (GSK2586881) has been tested in Phase 2 clinical trials for the potential treatment of acute respiratory distress syndrome, but it is not yet established if the compound will reduce viral load by acting as a decoy

Classically, the viral life cycle can be divided into six elements:‐ cell attachment, cell entry, viral uncoating, nucleotide replication, viral assembly and release (see Figure 1). Positive‐stranded RNA viruses replicate in the cytoplasm of infected cells, in close contact with intracellular membranes. This organization allows a concentration of viral and host factors to enable virus production and to evade innate immune responses (reviewed by Yager & Konan, 2019).

The SARS‐CoV‐2 coronavirus 30‐kb genome has 15 open reading frames (ORFs), two of which encode viral polyproteins that generate 16 non‐structural proteins (see below) (Wu et al., 2020). Historically, therapeutic benefit has been gained through exploitation of the differences between viral and host proteins that subserve superficially similar functions (proteases and nucleotide polymerases, for example). The rapidity with which structural elements of the SARS‐CoV‐2 proteome have been identified provides hope that drug discovery approaches will soon provide agents to target the virus selectively, with minimal impact on the host. Based on the evidence from orthologous proteins from other betacoronaviruses and the information currently available on SARS‐CoV‐2 (some of it not yet from peer‐reviewed sources), we propose here the priority targets for pharmacological investigation. That should not be taken to mean that research should be limited to these targets, since there are undoubtedly a number of functions of the viral proteins still to be ascertained. It would be remiss not to conduct a thorough examination of all the viral proteome, both in isolation and in combination. The strategies we learn from investigation of the host:viral interaction from SARS‐CoV‐2 will stand us in good stead for future viral threats.

### 2.1 Cellular attachment and entry: replication, assembly and release

Coronavirus binds to cell surface proteins on target cells and, following proteinase priming of spike proteins on the virus surface, the virus is internalized into endosomal fractions that are subsequently acidified or accumulates through a non‐endosomal route (Fehr & Perlman, 2015) (Figure 1). The endosomal route appears to involve clathrin (Inoue et al., 2007), but there are contradictory reports of the importance of the intracellular C‐terminus of ACE2 in this mechanism (Haga et al., 2008; Inoue et al., 2007). A fusion domain permits insertion of a key protein (the spike glycoprotein of the virus, S, see below), which then allows mixing of the viral and cellular membranes and subsequent release of the coronaviral genome into the cytoplasm.

Following entry into the host cell cytoplasm and viral uncoating, the replicase gene of the viral RNA is translated. The genome of coronaviruses consists of a single, continuous and linear ssRNA, capped at the 5′ end and with a 3′‐polyA tail (Fehr & Perlman, 2015). Translation occurs from ORF1a and 1b at the 5′ terminus, with a ribosomal frameshifting mechanism allowing the overlap between *ORF1a* and *ORF1b* to generate the two polyproteins pp1a and pp1ab (Fehr & Perlman, 2015; Perlman & Netland, 2009; Snijder et al., 2003; Thiel et al., 2003). In SARS‐CoV‐2, the polyproteins are long, 4,405 and 7,096 amino acids respectively. Encoded within the polyproteins of betacoronaviruses are two proteinases, papain‐like proteinase (PL_pro_) and chymotrypsin‐like proteinase (3CL_pro_). In SARS‐CoV, PL_pro_, derived from the polyproteins, has three endoproteinase target sites, which release nsp1–3 (Thiel et al., 2003). 3CL_pro_ has 11 cleavage sites to release the remaining non‐structural proteins. In the coronavirus family, these proteinases process the polyproteins to generate 16 functional non‐structural proteins identified as nsp1–16 (Anand, Ziebuhr, Wadhwani, Mesters, & Hilgenfeld, 2003; Cui et al., 2019; Kindler, Thiel, & Weber, 2016; Thiel et al., 2003; Ziebuhr et al., 2007).

**FIGURE 2:**
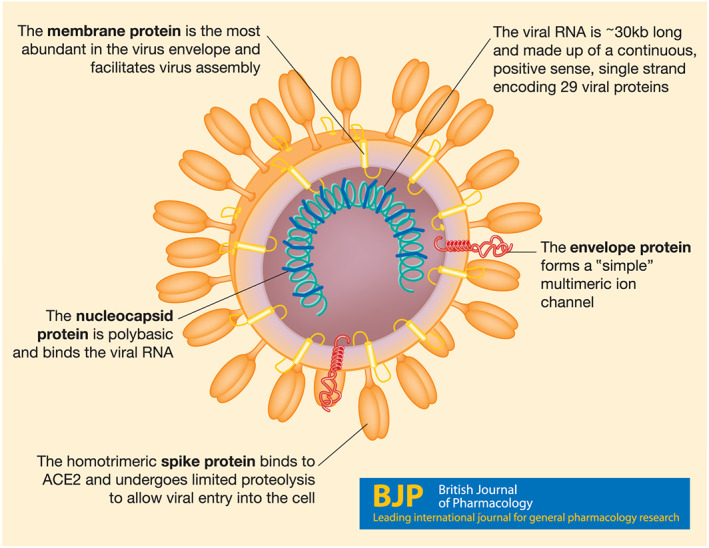
A cartoon of the virus structure, identifying the four structural proteins and the viral genome

Downstream of the ORF1a and 1b are genes encoding four structural proteins (spike, envelope, membrane and nucleocapsid) (Figure 2) and a short series (described as at least 13 in total, Srinivasan et al., 2020) of other proteins (see below). Once sufficient protein and RNA accumulate, coronavirus assembly takes place, centred on the structural proteins. The release of coronavirus particles involves the secretory pathway of the endoplasmic reticulum and Golgi apparatus and vesicular exocytosis (for review, see de Haan & Rottier, 2005; Fehr & Perlman, 2015) and it is likely, but as yet unconfirmed, that SARS‐CoV‐2 also adopts this mechanism.

To date, there is more evidence about the molecular detail involved in (and the possibilities to modify) viral recognition, entry and replication compared to uncoating, assembly and release, hence the attention paid here to the former three mechanisms.

## 3 TARGETING VIRUS RECOGNITION AND CELLULAR ENTRY

### 3.1 The cell‐surface anchor ‐ ACE2

Among the coronaviruses, the spike protein interacts with proteinases to anchor on host cell surfaces. The cell‐surface anchoring point for the alphacoronavirus HCoV‐229E is aminopeptidase N (also known as CD13, Yeager et al., 1992). For the betacoronavirus MERS‐CoV, dipeptidylpeptidase 4 (also known as CD26, Raj et al., 2013) is an anchor. Analysis of the co‐crystal structure suggested that the SARS spike protein binds to the active site of ACE2 (Li, Li, Farzan, & Harrison, 2005). Binding of SARS‐CoV spike to ACE2 seems to require cholesterol‐rich rafts in the host cells (Glende et al., 2008). Recent evidence points to the spike protein of SARS‐CoV‐2 also binding to ACE2. Both SARS‐CoV (Li et al., 2003) and SARS‐CoV‐2 (Hoffmann et al., 2020; Letko, Marzi, & Munster, 2020) have been described to require ACE2 to enter cells (Figure 1). A particular domain of the spike protein of SARS‐CoV‐2, a so‐called receptor‐binding domain (RBD), has been shown to facilitate binding to ACE2 (Hoffmann et al., 2020). The ACE2 peptidase active site is located remotely from the cell membrane (Li et al., 2005; Wrapp et al., 2020; Yan et al., 2020), into which the spike protein binds. The receptor‐binding domain of the spike protein is located in the S1 ectodomain, approximately a third of the way along the protein. ACE2 is a carboxypeptidase, which means it removes the terminal amino acid from oligopeptides, and so it seems unlikely that the spike protein is a substrate for ACE2.

In SARS‐CoV‐infected mouse lung, ACE2 protein expression was down‐regulated compared to uninfected mice (Kuba et al., 2005). Following SARS‐CoV spike protein administration to mice, angiotensin II was increased in the lungs (Kuba et al., 2005). These observations led to the suggestion that this was the molecular mechanism for the frequent development of acute respiratory distress syndrome (ARDS) during SARS‐CoV infections (Imai et al., 2005; Kuba et al., 2005).

ACE2 has been reported to be released from plasma membranes by proteolysis, thought to be through the action of TNFα convertase (ADAM17, a disintegrin and metalloproteinase domain containing protein 17, Lambert et al., 2005) (Figure 1). The activity of ADAM17 can be increased by GPCR activation, including the AT_1_ angiotensin receptor (Schafer, Marg, Gschwind, & Ullrich, 2004). ACE2 and ACE activities can be measured in human plasma (Herath et al., 2007; Lew et al., 2008; Ocaranza et al., 2006). Human plasma ACE2 activity is reported to be 'masked' by the presence of endogenous inhibitors (Lew et al., 2008), which do not yet appear to have been precisely defined. Blood ACE2 activity can be altered in pathology; for example, serum ACE2 was found to be decreased in patients following acute ischaemic stroke (Bennion et al., 2016).

The expression of ACE2 mRNA and enzyme activity in cardiac tissues were increased following repeated p.o. administration of the AT_1_ receptor antagonist losartan, while p.o. administration of an ACE inhibitor lisinopril only increased cardiac mRNA expression, but not enzyme activity (Ferrario et al., 2005).

Studies using disruption of the *ace2* gene in mice indicated an increase in circulating angiotensin II levels and a severe cardiac contractility defect, which could be 'rescued' with simultaneous genetic disruption of ACE (Crackower et al., 2002). An early investigation of *ACE2* polymorphisms in man failed to show an association with hypertension (Benjafield, Wang, & Morris, 2004). A study of SARS victims and *ACE2* polymorphisms failed to find a correlation with patient outcomes (Chiu et al., 2004).

### 3.2 The coronaviral spike protein

The spike protein is the largest viral structural protein (~1,200–1,400 amino acids) and is heavily glycosylated, forming extended trimeric structures providing the characteristic “crown” feature of coronaviruses (Belouzard, Millet, Licitra, & Whittaker, 2012) (see Figure 2). The ectodomain is divided into the S1 domain responsible for binding to ACE2, whereas the S2 domain is responsible for the fusion machinery. Following binding of the S1 domain to ACE2, a deformation of the pre‐fusion trimer results (Wrapp et al., 2020). Surface plasmon resonance of the binding of human ACE2 to the immobilized SARS‐CoV‐2 indicated an affinity (*K*_D_ value) of 15 nM, an order of magnitude larger than SARS‐CoV binding to ACE2 (Wrapp et al., 2020). Using a related label‐free technique, biolayer interferometry, affinities of 5 and 1.2 nM for binding of SARS‐CoV and SARS‐CoV‐2 spike protein, respectively, to human ACE2 has been reported (Walls et al., 2020).

Although a proteolytic cleavage site at the S1/S2 boundary of the SARS‐CoV spike protein is best characterized, a second site upstream of the fusion peptide in the S2 domain, called S2′, has also been described (Belouzard, Chu, & Whittaker, 2009). This raises the possibility that multiple other proteases might be targeted to influence coronavirus activation (Millet & Whittaker, 2015). A key difference between the spike proteins in SARS‐CoV and SARS‐CoV‐2 is the presence in the latter of a site at the S1/S2 boundary predicted to be sensitive to the proteinase furin, and which may be targeted during viral assembly and maturation (Walls et al., 2020).

The SARS‐CoV S2 domain has a pair of α‐helices, which may participate in coiled:coil structures during membrane fusion (Petit et al., 2005). The host complex of ZDHHC9 (Link to UniProt) with GOLGA7 (Link to UniProt), a palmitoyltransferase, which modifies the low MW G proteins NRAS and HRAS (Swarthout et al., 2005), also palmitoylates the cysteine‐rich S2 endodomain of the SARS‐CoV to facilitate membrane fusion (Petit et al., 2007).

Very recently, in a comparison of the S2 domains of SARS‐CoV and SARS‐Cov‐2, an enhanced capacity of the novel virus' S2 domain for membrane fusion was observed and suggested to result from eight differing amino acids (Xia et al., 2020). Using a series of oligopeptides conjugated to lipid entities, high affinity (IC_50_ values in the nanomolar range) inhibitors of cell fusion were identified.

### 3.3 Interfering with the ACE2:spike protein interaction

Given that the spike protein binds to the active site of ACE2 (Li et al., 2005), in theory, any alteration in the availability of the active site should influence the binding of the spike protein and, hence, interfere with SARS‐CoV‐2 infection. One option would be to provide an excess of an endogenous peptide substrate, or more conventionally to apply a selective enzyme inhibitor.

#### 3.3.1 Endogenous substrates of ACE2

ACE2, discovered in 2000 (Donoghue et al., 2000), shares 40% sequence similarity to ACE within the *N*‐terminal domain and is a type I transmembrane metallopeptidase. Unlike ACE, it functions as a zinc carboxypeptidase to cleave single C‐terminal amino acids from peptides, particularly hydrolysing Pro‐Phe residues in angiotensin‐(1–8) to angiotensin‐(1–7), [Pyr^1^]‐apelin 13 to [Pyr^1^]‐apelin‐(1–12) and [des‐Arg^9^]‐bradykinin to bradykinin‐(1–8) with high efficiency. It may also cleave other peptides less effectively (Vickers et al., 2002), shown below:

**Table:** 

Angiotensin I	⇨	angiotensin‐(1–9) + Leu
Angiotensin II	⇨	angiotensin‐(1–7) + Phe
Apelin‐(1–13)	⇨	QRPRLSHKGPMP + Phe
Apelin‐(1–36)	⇨	…. QRPRLSHKGPMP + Phe
[Des‐Arg^9^]‐Bradykinin	⇨	RPPGFSP + Phe
Dynorphin A‐(1–13)	⇨	YGGFLRRIRPKL + Lys

115 other peptides were not hydrolysed by ACE2 including adrenocorticotrophic hormone, calcitonin, cholecystokinin, met‐enkephalin, glucagon, glucagon‐like peptide‐1, melanin‐concentrating hormone, pituitary adenylyl cyclase‐activating polypeptide, somatastatin‐14, urocortin or vasoactive polypeptide (Vickers et al., 2002).

In humans, levels of mRNA encoding ACE2, together with immunoreactive peptide, are highest in the gastrointestinal tract, followed by heart, kidney, testes and gall bladder, and other tissues (Uhlen et al., 2015). Within organs, ACE2 immunoreactivity was predominantly localized to epithelial (for example, in the lungs) and endothelial cells from all vascular beds examined (Yang et al., 2017). Importantly, the ACE2 antisera used in this study for immunocytochemistry was the same as that employed in the study described in Section 3.3.3 (Hoffmann et al., 2020), to block entry of the virus in cell culture. The epitope of this antisera would be a rational starting point for the development of selective therapeutic antibodies.

The presence of ACE2 on airway epithelial cells is consistent with the isolation of SARS‐CoV‐2 from broncho‐alveolar lavage of patients with COVID‐19 and the infection of cultured airway epithelial cells (Zhu et al., 2020). In humans, levels of ACE2 immunoreactivity tend to be low. However, in addition to being up‐regulated by ACE inhibitors and angiotensin receptor antagonists (see above), ACE2 expression has been reported to be increased in human cardiovascular disease, for example, in the cardiomyopathic heart (Zisman et al., 2003). Since ACE2 is critical for viral entry, it may be one explanation for the high incidence of co‐morbidity of COVID‐19 patients with cardiovascular disease.

#### 3.3.2 Manipulation of ACE2 activity by synthetic agents

Assays employing fluorogenic surrogate substrates to screen for inhibitors of ACE2 activity are well‐established, for example, using methoxycoumarin‐RPPGFSAFK (Dnp)‐OH (Bennion et al., 2016; Ocaranza et al., 2006) or methoxycoumarin‐APK (Dnp)OH (Herath et al., 2007; Lew et al., 2008; Mores et al., 2008). Detailed protocols for the use of methoxycoumarin‐APK (Dnp)OH have been described for FRET‐based high throughput screening (Sriramula, Pedersen, Xia, & Lazartigues, 2017; Xiao & Burns, 2017).

This style of assay identified that ACE2 was not inhibited in the presence of 10μM lisinopril, enalaprilat or captopril, inhibitors of ACE (Tipnis et al., 2000). There are no licensed drugs described to inhibit ACE2 activity. However, DX600 is a peptide‐based ACE2 inhibitor (Huang et al., 2003), while MLN4760 and compound 28 are described as sub‐nanomolar potency ACE2 inhibitors (Mores et al., 2008).

There is evidence for allosteric regulation of ACE2 activity, in that a xanthenone derivative (XNT) was observed to enhance ACE2, but not ACE, activity in vitro with a potency of 20 μM (Hernandez Prada et al., 2008). An *in silico* study later identified a binding site in an allosteric hinge region of ACE2, distinct from the proteinase active site, against which 1,217 FDA‐approved drugs were screened (Kulemina & Ostrov, 2011). A subsequent kinetic assay with the recombinant enzyme and a fluorigenic substrate identified labetalol and diminazene as agents able to double the maximal velocity of ACE2 enzyme activity.

Whether any of these compounds alter the binding of the spike protein from either SARS‐CoV or SARS‐CoV‐2 or viral infection in general does not appear to have been examined yet.

A speculative area that should be explored further is the concept of enhancing the activity of the serine proteinase ADAM17 to increase cleavage and release of ACE2 from the membrane. Peptides such as angiotensin II are reported in animal models to cause release (“shedding”) following binding to AT_1_ receptors (Xu et al., 2017). Although angiotensin II is licensed by the Federal Drug Administration to treat sepsis (known as Giapreza, Davenport, Scully, de Graaf, Brown, & Maguire, 2020), it would be inadvisable as a treatment for COVID‐19 given the detrimental action of angiotensin II on the lungs. In contrast, the investigational agent [Pyr^1^]‐apelin‐13 is currently used in clinical studies (Davenport et al., 2020) and may also interact with its cognate receptor to down‐regulate membrane‐expressed ACE2. This peptide also has beneficial effects on the heart, including an increase in cardiac output (Japp et al., 2010).

#### 3.3.3 Using biopharmaceutical/antibody approaches to target ACE2:spike interactions

An alternative approach to the small molecule manipulation of the ACE2 enzyme would be to target the spike or ACE2 proteins with selective antibodies. Antibodies directed against ACE2 led to a reduction in SARS‐CoV‐2 virus entry into target cells (Hoffmann et al., 2020), although this is likely to be some distance away from a therapeutic application.

A truncated version of human recombinant ACE2, lacking the transmembrane domain, mitigated against SARS‐CoV infection of cells (Li et al., 2003) and has been used in animal models to reduce symptoms of severe acute lung failure (Imai et al., 2005), diabetic nephropathy (Oudit et al., 2010), and cardiac hypertrophy and fibrosis (Zhong et al., 2010). Treating SARS‐CoV‐2 victims with a soluble form of ACE2 (Batlle, Wysocki, & Satchell, 2020) or a fusion protein of the spike‐binding portion of ACE2 combined with the Fc portion of human IgG (Lei et al., 2020) has been suggested.

Apeiron Biologics has approval to conduct a Phase II clinical trial of APN01 (human recombinant ACE2) for the treatment of COVID‐19 in three European countries (Austria, Germany and Denmark) (NCT04335136). This recombinant version of ACE2 was originally licensed to GlaxoSmithKline and previously tested as GSK2586881 in a Phase 2 multicentre trial (NCT01597635) in patients with lung injury or acute respiratory distress syndrome, both features of SARS and MERS (and now COVID‐19). The study tested the hypothesis that cleavage of angiotensin II (which causes lung injury—vasoconstriction, inflammation, fibrosis, vascular leak and sodium absorption) to angiotensin‐(1–7) would have counter regulatory beneficial action and reduce long term injury. GSK2586881 was well‐tolerated in patients with acute respiratory distress syndrome and the rapid modulation of peptides of the renin‐angiotensin system demonstrated target engagement, in that levels of angiotensin II decreased rapidly, whereas angiotensin‐(1–7) levels increased and remained elevated for 48 h, although the study was not powered to detect changes in acute physiology or clinical outcomes (Khan et al., 2017).

Sera from convalescent SARS‐CoV patients prevented the cell entry of SARS‐CoV‐2 (Hoffmann et al., 2020) and this approach has been used with some success in the SARS, MERS and COVID‐19 outbreaks (for review, see Bloch et al., 2020). The difficulty in identifying the precise molecular mechanism/s of convalescent sera action and issues with collection, standardization and scaling‐up will be a challenge (Bloch et al., 2020).

A bacterial equivalent of ACE2 (based on 3D structure rather than primary sequence) termed B38‐CAP has been described, which is reported to reduce hypertension and limit cardiac dysfunction in an animal model (Minato et al., 2020). Whether this agent might provide a decoy anchor to “chelate” viral particles prior to cell entry has not been investigated.

In a preliminary (as yet, not peer reviewed) study, a conformational change in the S1 receptor‐binding domain of the SARS‐CoV‐2 spike protein in the presence of heparin was noted (Mycroft‐West et al., 2020). Cell‐surface heparan sulphate glycosaminoglycans have previously been suggested to be a lactoferrin‐sensitive alternative attachment point for the SARS‐CoV virus (Lang et al., 2011). These observations suggest further routes for pharmacological targeting of viral infection and propagation.

### 3.4 The cell‐surface priming mechanism ‐ TMPRSS2

TMPRSS2 is a single transmembrane domain protein with an extracellular serine protease domain, which appears to cleave substrates preferentially at basic residues (arg/lys), with a calcium‐binding LDL receptor class A domain (Paoloni‐Giacobino, Chen, Peitsch, Rossier, & Antonarakis, 1997). The *TMPRSS2* gene encodes a cell‐surface proteinase (transmembrane serine protease 2, TMPRSS2) and is located at chromosomal locus 21q22.3 in close proximity to *ERG*, a gene encoding an ETS transcription factor (Link to UniProt, Paoloni‐Giacobino et al., 1997) (*ERG* fusion with *EWS* leads to Ewing's sarcoma). Fusion of the *TMPRSS2* and *ERG* (or the related *ETV1*) genes has been reported to occur in the majority of prostate cancers and is suggested to lead to an androgen‐dependent amplification of ETS‐regulated genes (Tomlins et al., 2005). TMPRSS2 expression is androgen‐regulated (Chen et al., 2019; Lin et al., 1999). It is expressed highly in prostate cancer (Lin et al., 1999; Lucas et al., 2008) (for review, see Tanabe & List, 2017) and loss of TMPRSS2 in the prostate is associated with reduced metastatic potential (Lucas et al., 2014). In aggressive versions of prostate cancer, TMPRSS2 undergoes autocatalytic proteolysis at Arg^255^‐Ile^256^ (Afar et al., 2001), where the two chains may remain in combination due to interchain disulphide bridges (Chen et al., 2010) or the catalytic moiety may be secreted (Chen et al., 2010). In LNCaP human prostate cancer cells, the PPARα (NR1C1) agonist fenofibrate was able to mitigate against the androgen receptor agonist‐evoked increase in TMPRSS2 expression (Zhao et al., 2013).

Following binding of the S protein to ACE2, TMPRSS2 “primes” the spike protein to facilitate entry of the virus into the target cell (Hoffmann et al., 2020; Matsuyama et al., 2020). Pathogenesis of two strains of influenza virus has been reported to be markedly diminished by gene disruption of *tmprss2* in mice (Hatesuer et al., 2013; Tarnow et al., 2014), inferring that targeting this enzyme may have antiviral potential.

#### 3.4.1 Interfering with the TMPRSS2:spike protein interaction

Using immunohistochemical analysis (Bertram et al., 2012) and, very recently, using single nuclei and single‐cell RNA sequencing (Lukassen et al., 2020), of lung samples from otherwise healthy subjects, ACE2 and TMPRSS2 were shown to be co‐expressed in human bronchial epithelial cells, which could be a nexus for primary infection. A similar approach identified co‐expression of ACE2 and TMPRSS2 in nasal goblet cells, lung type II pneumocytes and small intestine absorptive epithelia (Ziegler et al., 2020). In the same study, human primary nasal epithelial cells showed an up‐regulation in ACE2 expression following 12h incubation with IFN‐α2 and IFN‐γ, which suggests the potential for a feed‐forward mechanism whereby the virus interacts preferentially with “activated” cells to suppress the innate immune response (see below) (Ziegler et al., 2020).

By analogy with the previous consideration of ACE2 (above), alternatives to manipulate TMPRSS2 activity would be to provide endogenous substrates or synthetic inhibitors. However, the potential to make use of endogenous substrates seems limited. Thus, although TMPRSS2 has been described to hydrolyse and activate the cell‐surface GPCR proteinase‐activated receptor 2 (Wilson et al., 2005), mice lacking *tmprss2* failed to display an overt phenotype (Kim, Heinlein, Hackman, & Nelson, 2006).

As with ACE2, there are no reports of licensed drugs which inhibit TMPRSS2 activity. Cbz‐GGR‐aminomethylcoumarin has been described as a surrogate fluorogenic substrate suitable for high‐throughput screening (Paszti‐Gere et al., 2016), although it is also a substrate for other proteinases, such as chymotrypsin. I432, a 3‐amidinophenylalanine, has been described as a high affinity selective inhibitor (compound 92, *K*_i_ of 0.9 nM) of TMPRSS2 (Meyer et al., 2013). In IPEC‐J2 pig jejunal epithelial cells, 10‐ to 50‐μM I432 reduced TMPRSS2‐derived product in cell media (Paszti‐Gere et al., 2016).

In an investigation of SARS‐CoV entry into HeLa cells expressing recombinant ACE2 and TMPRSS2, a number of serine proteinase inhibitors (benzamidine, aprotinin, gabexate, tosyl lysyl chloromethyl ketone and camostat) were tested (mostly) at 10 μM for 30 min before exposure to pseudotyped viruses. Only camostat was effective at reducing viral entry (Kawase, Shirato, van der Hoek, Taguchi, & Matsuyama, 2012) and further experiment suggested that 1 μM camostat was also effective, but only when TMPRSS2 was expressed. At 10 and 50 μM, camostat inhibited cell entry of the SARS‐CoV and SARS‐CoV‐2 spike protein (Hoffmann et al., 2020). A direct inhibition of TMPRSS2 activity appears not to have been reported for camostat.

### 3.5 Potential ancillary proteins for virus entry ‐ B^0^AT1/SLC6A19 and B^0^AT3/SLC6A18

The SLC6 family of transporters includes the well‐characterized NET, SERT and DAT monoamine transporters, as well as the less well‐exploited neutral amino acid transporter subfamily. B^0^AT1/SLC6A19 and B^0^AT3/SLC6A18 allow sodium‐ and chloride‐dependent accumulation of neutral, aliphatic amino acids at the apical membranes of epithelial cells in the small intestine (B^0^AT1/SLC6A19) and kidney (B^0^AT1/SLC6A19 and B^0^AT3/SLC6A18) (for review, see Broer & Gether, 2012). B^0^AT3/SLC6A18 is also highly expressed in the GI tract and gall bladder (Protein Atlas). The cell‐surface expression of these neutral amino acid transporters is dependent on co‐expression of ACE2 (Fairweather, Broer, O'Mara, & Broer, 2012; Kowalczuk et al., 2008), aminopeptidase N (Fairweather et al., 2012) or collectrin (an adaptor protein, which has high homology to the transmembrane region of ACE2, Camargo et al., 2009, Link to UniProt), in an apparently tissue‐dependent manner (Kuba, Imai, Ohto‐Nakanishi, & Penninger, 2010). A recent cryo‐EM structure suggested that ACE2 and B^0^AT1/SLC6A19 form a heterodimer, which pairs up through interfaces between the two ACE2 partners (Figure 1), with the receptor‐binding domain of SARS‐CoV‐2 spike protein binding to the peptidase active site of ACE2 (Yan et al., 2020) suggesting that B^0^AT1/SLC6A19 may facilitate entry of the novel coronavirus. In the small intestine, absorptive epithelial cells were identified to co‐express mRNAs encoding for ACE2 and TMPRSS2 (Ziegler et al., 2020). Although it is not yet tested, it would be attractive to speculate that the co‐localized expression of these targets may play a role in the faecal:oral transmission of coronavirus (Yeo, Kaushal, & Yeo, 2020).

#### 3.5.1 Interfering with the neutral amino acid transporters

Assays for B^0^AT1/SLC6A19 and B^0^AT3/SLC6A18 tend to be traditional accumulation of amino acids labelled with ionizing or stable isotopes. Recently, a primary screen using a membrane potential‐sensitive fluorescence‐based assay was used and followed up with a stable isotope accumulation assay to identify a novel inhibitor, cinromide, which exhibited modest potency (0.3–0.4 μM) for inhibiting B^0^AT1/SLC6A19 in cell‐based assays (Danthi et al., 2019).

## 4 TARGETING VIRAL UNCOATING AND REPLICATION

### 4.1 Viral uncoating

Once inside the cell, the endosomal cysteine proteases cathepsin B and cathepsin L have been described to process SARS‐CoV (Simmons et al., 2005) and this appears to be maintained for SARS‐CoV‐2 (Hoffmann et al., 2020), although the significance of such intracellular proteinase activity is unclear. Potent inhibitors for these two proteinases have been reported as pharmacological probes, but there are no licensed drugs identified to target them.

Following entry into the cell, many viruses accumulate in acidified lysosome‐like vesicles and so weak bases (including ammonium chloride and chloroquine), which target the lysosome have been used in vitro to target this mechanism. Ammonium chloride (at 20 mM) has been described as a non‐specific inhibitor of viral replication in vitro, targeting viral uncoating (Mizzen, Hilton, Cheley, & Anderson, 1985) and, at 50 mM, ammonium chloride inhibited cell entry of both SARS‐CoV and SARS‐CoV‐2 (Hoffmann et al., 2020). Chloroquine was also observed to reduce infection of L cells by mouse hepatitis virus 3 (Krzystyniak & Dupuy, 1984).

### 4.2 Viral replication

Following entry into the cell, the virus subverts nucleotide, protein, lipid, and carbohydrate turnover of the host cell to produce multiple copies of itself. The viral RNA is translated into multiple proteins to produce the replication machinery. As protein translation from the viral genome occurs, the two polyproteins are the first to be synthesized, with the two intrinsic proteases able to cleave the polyproteins into their constituent products.

#### 4.2.1 Targeting the viral proteinases

The low sequence similarities between mammalian and viral proteases has allowed successful drug targeting of viral diseases, including both HIV/AIDS and HCV/hepatitis C. The genome of SARS‐CoV‐2 contains two proteinases intrinsic to the polyproteins, PL_pro_ and 3CL_pro_.

### 4.2.1.1 The papain‐like proteinase of the virus (PL _pro_)

The more *N*‐terminally located PL_pro_ is the larger (~2,000 aa) of the two proteins (for review, see Baez‐Santos, St John, & Mesecar, 2015; Lei, Kusov, & Hilgenfeld, 2018) and in SARS‐CoV is a membrane‐associated, polyfunctional entity (Harcourt et al., 2004). Sequence modelling of SARS‐CoV‐2 PL_pro_ suggested the presence of 6 TM domains towards the C‐terminus, with the majority of the protein extending into the cell cytoplasm (Angeletti et al., 2020). In other coronaviruses, the enzyme is also capable of hydrolysing ubiquitin from protein substrates (Barretto et al., 2005; Ratia et al., 2006), as well as removing the ubiquitin‐like protein IFN‐stimulated gene 15 (ISG, Link to UniProt) from ISG‐conjugated proteins (Yang et al., 2014). Using the orthologous proteinase from the mouse hepatitis coronavirus, analysis of three distinct structural domains suggested that the PL_pro_ domain coincided with the deubiquitinylating and deISGylating functions (Chen et al., 2015). In SARS‐CoV, the PL_pro_ also contains an ADP‐ribose‐1″‐phosphatase functional phosphatase domain directed at ADP‐ribose‐1″‐phosphates, although the functional significance of the hydrolase activity may be less impactful than the capacity to bind ADP‐ribose, at least for the enzyme from HCoV‐229E (Putics, Filipowicz, Hall, Gorbalenya, & Ziebuhr, 2005). This domain is thought to contribute to processing of the viral subgenomic RNAs and the suppression of the innate immune system through reducing IFN production (Lei et al., 2018).

Investigating the peptidase activity of SARS‐CoV PL_pro_ suggested a preference for larger proteins (ubiquitinated or ISGylated) rather than simpler fluorescent‐tagged oligopeptide substrates (Baez‐Santos, Mielech, Deng, Baker, & Mesecar, 2014; Lindner et al., 2005; Lindner et al., 2007; Ratia, Kilianski, Baez‐Santos, Baker, & Mesecar, 2014) making screening more complicated.

### 4.2.1.2 The chymotrypsin‐like proteinase, 3C‐like proteinase of the virus (3CL _pro_)

The smaller proteinase from SARS‐CoV‐2 is 3CL_pro_ (sometimes called the main prote(in)ase, M_pro_). The use of *i* *n silico* docking models of SARS‐CoV‐2 3CL_pro_ has led to suggestions that particular existing antiviral agents, including velpatasvir and ledipasvir (licensed agents for treating hepatitis C when combined with sofosbuvir in the United Kingdom), should be screened for functional activity (Chen, Yiu, & Wong, 2020). A recent screen of ~10,000 compounds including approved drugs, candidate drugs and natural products used a substrate derived from the *N*‐terminal autocleavage site of the SARS‐CoV‐2 3CL_pro_, which was modified (methylcoumarinylacetyl‐AVLQSGFR‐Lys (Dnp)‐Lys‐NH_2_) to allow a FRET‐based assay (Jin et al., 2020). The same substrate was used in a screen of the equivalent enzyme from another coronavirus, HCoV‐HKU1, which transferred to humans (Zhao et al., 2008).

A number of inhibitors of the SARS‐CoV 3CL_pro_ proteinase have been described (Goetz et al., 2007; Lu et al., 2006; Yang et al., 2006), without progressing into the clinic. Recently, an *in silico* approach using orthologues of the SARS‐CoV 3CL_pro_ from other coronaviruses and enteroviruses allowed production and testing in vitro of a series of α‐ketoamides (Zhang et al., 2020). One compound (11r) exhibited submicromolar potency against SARS‐CoV 3CL_pro_ in a cell‐free FRET‐based assay and micromolar potency in a cell infection assay with SARS‐CoV (Zhang et al., 2020).

In a very recent report, the SARS‐CoV‐2 3CL_pro_ expressed in HEK293 cells was found to interact with histone deacetylase 2 (HDAC2) by affinity purification/MS (Gordon, Jang, et al., 2020). A number of approved drugs target HDAC2 in the treatment of various T cell lymphomas, including romidepsin, belinostat and vorinostat with nanomolar potency (Bradner et al., 2010).

#### 4.2.2 Targeting nucleotide turnover

A relatively large proportion of the viral genome is inevitably devoted to nucleotide turnover. For SARS‐CoV‐2, this includes nsp7/nsp8/nsp12 as an RNA‐dependent RNA polymerase, nsp13 as a helicase, nsp10/nsp14 as an 3′‐to‐5′ exonuclease complex, nsp15 as an endoribonuclease and nsp16 as a 2′‐*O*‐ribose methyltransferase.

Remdesivir (currently in clinical trials to treat COVID‐19) is described as a non‐selective inhibitor of multiple RNA viruses and has shown some efficacy in MERS‐CoV and SARS‐CoV infection of monkeys (de Wit et al., 2020). In in vitro investigations, the triphosphate analogue of remdesivir inhibited RNA synthesis of MERS‐CoV RNA‐dependent RNA polymerase (primarily nsp8/nsp12 complexes derived from co‐expression in insect cells of a construct containing nsp5, nsp7, nsp8, and nsp12) with an IC_50_ value of 32 nM when nucleotide levels were low, increasing to 690 nM at higher nucleotide concentrations (Gordon, Tchesnokov, Feng, Porter, & Gotte, 2020). *In silico* modelling identified that remdesivir, as well as the approved antiviral drugs ribavirin, sofosbuvir, and tenofovir, could bind tightly to the active site of nsp12 from SARS‐CoV‐2, based on the crystal structure of SARS‐CoV (Elfiky, 2020).

However, ribavirin alone had no significant effect in a clinical trial with SARS patients, although a combination of ribavirin with lopinavir–ritonavir and corticosterone had a lower rating of acute respiratory distress syndrome and death (for review, see Zumla et al., 2016). In‐depth analysis has not been completed with MERS patients, although an ongoing Phase 2 clinical trial for MERS uses a combination therapy of lopinavir/ritonavir and IFN‐β1b (Arabi et al., 2020).

Nsp13 is a helicase, which enables unwinding of duplex RNA. The exoribonuclease activity of nsp14 sets the coronaviruses apart (Snijder et al., 2003), as the enzyme is suggested to remove damaging mutations from the genome (Eckerle et al., 2010; Sevajol, Subissi, Decroly, Canard, & Imbert, 2014). In other coronaviruses, the endoribonuclease nsp15 has some selectivity for hydrolysing polyU sequences (Hackbart, Deng, & Baker, 2020). This enables the virus to delay or minimize initiation of the innate immune system by hydrolysing negative sense polyU nucleotides, which activate the MDA5 system to evoke intereferon production (discussed further below). Nsp16 is a methyltransferase, which uses S‐adenosyl‐L‐methionine as a co‐substrate to assist in cap formation (Decroly et al., 2008).

### 4.2.2.1 Protein:protein interactions in recombinant expression

In a very recent report, a series of tagged recombinant proteins from SARS‐CoV‐2 were expressed in HEK293 cells and then protein partners were identified by affinity purification/MS (Gordon, Jang, et al., 2020). For nsp12 (RNA‐dependent RNA polymerase) and nsp14 (3′‐5′‐exonuclease) of SARS‐CoV‐2, interactions with receptor interacting protein kinase 1 (RIPK1) and inosine monophosphate dehydrogenase 2 (IMPDH2) respectively, were identified. For these two targets, there are established approved drugs. Thus, ponatinib, which is used to treat acute myelogenous leukaemia or chronic myelogenous leukaemia (Philadelphia chromosome), targets multiple protein kinases, inhibiting RIPK1 with an IC_50_ value of 12 nM (Najjar et al., 2015). Mycophenolic acid and ribavirin are IMPDH2 inhibitors with IC_50_ values of 20 nM (Nelson, Eugui, Wang, & Allison, 1990) and 1‐3 μM (Wittine et al., 2012) ranges, respectively, with clinical uses in organ transplantation and antiviral therapy, respectively.

Reservations about the use of ribavirin have already been noted above. Mycophenolic acid as a monotherapy was examined in a MER‐CoV‐infected non‐human primate model, where the authors concluded it actually worsened the condition (Chan et al., 2015).

Nsp13 (helicase) and nsp15 (endoribonuclease) have been described to bind to centrosome‐associated protein 250 (CEP250) and RNF41 (also known as NRDP1, Link to UniProt) respectively, in a report of recombinant expression (Gordon, Jang, et al., 2020). CEP250 is suggested to influence centrosome cohesion during interphase (de Castro‐Miro et al., 2016) and to be elevated in peripheral T cell lymphomas (Cooper et al., 2011). The functional relevance of nsp13 interaction with CEP250 is not yet clear. RNF41 is an E3 ubiquitin ligase, which polyubiquitinates myeloid differentiating primary response gene 88 (MyD88, link to UniProt), an adaptor protein for Toll‐like receptors, which allows activation of TBK1 and IRF3 (see below) and thereby increases type I IFN production (Wang et al., 2009).

#### 4.2.3 Targeting phospholipid turnover

The lipid profile of viruses appears to be important in terms of viral entry into the cell, either as sites for anchoring or for endocytosis (for review, see Heaton & Randall, 2011; Mazzon & Mercer, 2014). Replication of SARS‐CoV is reported to take place associated with the endoplasmic reticulum in “replicative organelles” incorporating convoluted membranes and interconnected double‐membrane vesicles, inferring a capacity for the virus to induce extensive reorganization of intracellular phospholipid membranes (Knoops et al., 2008). Three non‐structural proteins from SARS‐CoV with transmembrane domains, nsp3 PL_pro_ (see above), nsp4 and nsp6 when co‐expressed in model cells prompted the formation of these double‐membrane vesicles (Angelini, Akhlaghpour, Neuman, & Buchmeier, 2013), although it is unclear whether specific catalytic activities are necessary for this action.

The lipidome of influenza virus (also a positive strand RNA virus) consists of glycerophospholipids, sterols (mainly cholesterol) and sphingolipids, with sphingolipids and cholesterol enriched compared to the host cell membrane (Gerl et al., 2012), but there does not yet appear to be a parallel investigation of SARS‐CoV.

Cytosolic phospholipase A_2α_ (cPLA_2_α) hydrolyses phospholipid to produce lysophospholipids and free fatty acids. Using alphacoronavirus HCoV‐229E‐infected Huh‐7 cells, inhibition of cPLA_2_α using pyrrolidine‐2 at higher concentrations (20 μM) evoked an inhibition of viral titre (Muller et al., 2018). Arachidonoyl trifluoromethylketone, a non‐selective inhibitor of multiple eicosanoid‐metabolizing enzymes including PLA_2_ isoforms, also inhibited viral titres at higher concentrations (Muller et al., 2018). Transmission electron microscopy suggested that cPLA_2_α inhibition reduced the density of double‐membrane vesicles (Muller et al., 2018). Analysis of lipid metabolites indicated that HCoV‐229E‐infected Huh‐7 cells showed increases in levels of ceramides, lysophospholipids and phosphatidylglycerols, with decreases in phosphatidic acids (Muller et al., 2018). 20‐μM pyrrolidine‐2 inhibited the elevations in lysophospholipids and phosphatidylglycerols, but not the ceramides. Intriguingly, some selectivity of the involvement of PLA_2α_ was suggested as pyrrolidine‐2 also displayed antiviral activities against other members of the *Coronaviridae* (and *Togaviridae*) families, while members of the *Picornaviridae* family were not affected.

Although speculative, there is the possibility that some of the benefits of glucocorticoid administration in the clinic might be the up‐regulation of annexins and the subsequent binding and concealment of membrane phospholipid from further metabolism (for review, see Lemmon, 2008). While clearly some distance from a validated target, targeting the host availability of key structural (phospho)lipids, particularly sphingolipids, has been proposed to be a useful strategy in preventing propagation of enveloped human RNA viruses, including influenza, HIV and hepatitis C (Yager & Konan, 2019). Currently, however, assays to screen inhibitors of cPLA_2_α are relatively limited.

#### 4.2.4 Targeting carbohydrate turnover

Given that a number of the viral proteins, including the two structural proteins spike and membrane, are glycoproteins, there is clearly a diversion of sugars from the host. It is unclear as yet whether specific sugars are involved and whether specific host glycosyltransferases are involved in the processing of coronavirus glycoproteins and might, therefore, form further tractable targets for drug discovery. Notably, in studies using site‐directed mutagenesis of the spike protein from SARS‐CoV, glycosylation was identified at three glutamine residues within the S1 region, with no loss of binding to ACE2‐expressing cell of mutated (non‐glycosylated) fragments (Chakraborti, Prabakaran, Xiao, & Dimitrov, 2005).

### 5 THE OTHER VIRAL STRUCTURAL PROTEINS

#### 5.1 The envelope protein E

The envelope proteins of SARS‐CoV, HCoV229E and MERS are small (<100 aa) single transmembrane domain proteins (see Figure 2) and constitute ion channels with selectivity for monovalent cations over monovalent anions (Wilson, McKinlay, Gage, & Ewart, 2004; Zhang et al., 2014) apparently forming homopentamers in model membranes (Pervushin et al., 2009; Surya, Li, Verdia‐Baguena, Aguilella, & Torres, 2015). Infecting or transfecting the coronavirus E message into cells results in accumulation of protein in the Golgi region (Ruch & Machamer, 2012). Conserved cys residues proximal to the transmembrane domain internally within the virus are palmitoylated (Lopez, Riffle, Pike, Gardner, & Hogue, 2008), a post‐translational modification suggested to allow an internal inflexion point in the protein (Ruch & Machamer, 2012).

Hexamethylene‐amiloride has been described as an inhibitor of the HIV‐1 virus Vpu ion channel (Ewart, Mills, Cox, & Gage, 2002) and to reduce virus proliferation in human macrophages in culture (Ewart et al., 2004). Hexamethylene‐amiloride, but not the clinically used amiloride, inhibited the SARS‐CoV envelope protein‐associated ion channel activity when expressed in HEK293 cells (Pervushin et al., 2009).

Amantadine has had multiple uses clinically, including in the therapy of Parkinson's disease (for review, see Vanle et al., 2018). It has been used to treat influenza A infection through targeting the M2 ion channel (Holsinger, Nichani, Pinto, & Lamb, 1994; Pinto, Holsinger, & Lamb, 1992; Wang, Takeuchi, Pinto, & Lamb, 1993), although it is no longer recommended in the United Kingdom or United States because of drug resistance (for review, see Li, Chan, & Lee, 2015). Amantadine at higher concentrations (100 μM) was found to inhibit the SARS‐CoV E protein expressed in model membranes (Torres et al., 2007).

SARS‐CoV E protein was identified as being pro‐apoptotic upon transfection into Vero E6 monkey epithelial cells, where it localized to both plasma membrane and punctate cytoplasmic locations (Chan et al., 2009). Indeed, the SARS‐CoV E protein's ion channel function has been linked to calcium entry into endoplasmic reticulum/Golgi membrane complexes and the subsequent activation of the NLRP3 inflammasome, leading to IL‐1β production (Nieto‐Torres et al., 2015).

siRNA targeting of the envelope protein of SARS‐CoV reduced virus release in culture media, without altering N and P gene expression in FRhK‐4 monkey kidney epithelial cells (Lu et al., 2006). A similar observation was reported for the ORF4a protein (derived from the *Orf4a* gene) of HCoV229E (Zhang et al., 2014). Infecting mice with SARS‐CoV in which the E protein ion channel function was disrupted showed unchanged viral proliferation but reduced IL‐1β and oedema levels in the lungs and better survival over 10 days post‐infection (Nieto‐Torres et al., 2014).

In a very recent report, the E protein of SARS‐CoV‐2 has been reported to interact with BRD2/BRD4 BET family bromodomain kinases when expressed in HEK293 cells (Gordon, Jang, et al., 2020). JQ1 and RVX208 are BRD2/4 inhibitors with IC_50_ values with 40‐120and 50‐1,800nM ranges respectively.

#### 5.2 The membrane protein M

The membrane protein is usually regarded as the most abundant protein in the coronavirus envelope (see Figure 2) and is of intermediate size in SARS‐CoV‐2 (222 aa). It is thought to assist in viral assembly by collating the other surface structural proteins (Ruch & Machamer, 2012).

#### 5.3 The nucleocapsid phosphoprotein N

The N protein is of moderate size in SARS‐CoV‐2 (419 aa), highly basic and binds the viral RNA as a dimeric entity (Fan et al., 2005) into nucleocapsids (see Figure 2), which afford protection for the viral genome, while also providing access for replication at appropriate times (for review, see McBride, van Zyl, & Fielding, 2014). In a very recent report, the N protein of SARS‐CoV‐2 was tagged and expressed in HEK293 cells and then protein partners were identified by affinity purification/MS (Gordon, Jang, et al., 2020). The N protein was suggested to interact with casein kinase 2 (CK2), La‐related protein 1 (LARP1, Link to UniProt) and stress granule protein Ras GTPase‐activating protein‐binding protein 1 (G3BP1, Link to UniProt). CK2 phosphorylates a broad range of cellular targets, mostly in the nucleus, to regulate development and differentiation (for review, see Gotz & Montenarh, 2017). Although not in use clinically, two inhibitors are described to target CK2 with high affinity. Silmitasertib is a CK2 inhibitor with an IC_50_ value of 1 nM (Pierre et al., 2011), while TMCB has a *K*_i_ value of 21 nM (Janeczko, Orzeszko, Kazimierczuk, Szyszka, & Baier, 2012). LARP1 is an RNA‐binding protein, which releases RNA when phosphorylated by mTORC1 (Fonseca et al., 2015; Hong et al., 2017). LARP1 seems to preferentially bind 5′‐terminal oligopyrimidines with an as‐yet unclear cellular role (Philippe, van den Elzen, Watson, & Thoreen, 2020). Of the three targets suggested to associate with SARS‐CoV‐2 N phosphoprotein, G3BP1 seems a relevant focus for therapy against COVID‐19. G3BP1 regulates the innate immune response (Kim, Sze, Liu, & Lam, 2019; Liu et al., 2019; Wiser, Kim, & Ascano, 2019; Yang et al., 2019) and stress granules reduce the replication of MERS‐CoV (Nakagawa et al., 2018), so there is a potential for targeted drug discovery.

### 6 INTERACTIONS WITH THE HOST INNATE IMMUNE SYSTEM

SARS‐CoV produces proteins that interfere with IFN pathways (nsp1, nsp3, nsp16, ORF3b, ORF6, ORF9b, M and N proteins Wong, Lui, & Jin, 2016) and NLRP3 inflammasome activators (E, ORF3a, and ORF8b), which are closely related to orthologues found in SARS‐CoV‐2. Fung, Yuen, Ye, Chan, and Jin (2020) have recently reviewed the molecular aspects whereby SARS‐CoV and, by inference SARS‐CoV‐2, evade immune surveillance, activate the inflammasome and cause pyroptosis. Other coronaviruses may give an indication as to how this is happening. HCoV‐229E rapidly kills dendritic cells, while monocytes are much more resistant. The rapid invasion of and replication in dendritic cells kills them within a few hours of infection (Mesel‐Lemoine et al., 2012). Dendritic cells are sentinel cells in the respiratory tract and plasmacytoid dendritic cells are a crucial antiviral defence via IFN production and by modifying antibody production. Thus, these viruses can impair control of viral dissemination and the formation of long‐lasting immune memory. Penetration of SARS‐CoV‐2 infection deep into the lungs and eventually the alveolae, results in the “cytokine storm”, which accompanies pneumonia and lung fibrosis and is probably a major determinant of the necessity for intubation and also mortality (Shi, Wang, et al., 2020). It is currently not known what specific factor/s differentiate the patients who develop this, although mortality among younger health workers may indicate that initial viral load may play a role. Immunological agents, which can prevent or control the “cytokine storm” could therefore have a major effect on necessity to intubate and mortality. Tocilizumab is a monoclonal antibody targeting IL‐6 receptors, as a means to interfere with the effects of chronic autoimmune disorders such as rheumatoid arthritis. The Chinese Clinical Trials Registry has two studies that are designed to evaluate tocilizumab efficacy in patients with severe COVID‐19 pneumonia (Registration Numbers ChiCTR2000029765 and ChiCTR2000030442). Similarly, anakinra, which is a slightly modified version of an endogenous antagonist of IL‐1 receptors, is being investigated in clinical trials in multiple locations in patients with COVID‐19 infection (NCT04324021, NCT04330638, and NCT02735707).

It has been reported that in stage III of COVID‐19, a critical point with a high viral load and severe respiratory involvement, lungs of patients appear with “ground‐glass” patches in CT scans, while autopsy reports indicate that the lungs are filled with a “clear liquid jelly” (Shi, Wang, et al., 2020; Xu et al., 2020), similar to an observation in drowning victims. On the hypothesis that inflammation‐driven hyaluronan production (via hyaluronan synthase 2 [HAS2] Link to UniProt) and associated water retention may be critical. A recent study proposed therapy via administration of recombinant hyaluronidase or inhibitors of HAS2 (Shi, Wang, et al., 2020).

The interaction between the virus and the innate immune system is complex and multifactorial, with temporal intricacies. It is beyond the scope of this review to identify all the multiple components and so we discuss here those pathways we consider most tractable.

### 6.1 Viral nucleotides and MDA5/MAVS/IFN production

The positive sense RNA of coronaviruses is translated to produce the replication machinery, which allows complementary negative sense RNA to be synthesized, which itself is the template for the synthesis of positive strand RNA. As a consequence, double‐stranded RNA is produced, which act as a pathogen‐associated molecular pattern (PAMP) targeting MDA5 (IFN induced with helicase C domain I, also known as melanoma differentiation antigen 5, Kato et al., 2006) from the RIG‐1‐like receptor family of cytoplasmic pattern recognition receptors (for reviews, see Schlee, 2013; Bryant et al., 2015). MDA5 differs from RIG‐1 (DexD/H‐box helicase 58, also known as retinoic acid‐inducible gene 1) in recognizing longer dsRNA (Goubau et al., 2014; Kato et al., 2006), and it has been proposed that this differentiates the sensing of positive‐stranded viruses by MDA5 compared to negative strand virus sensing by RIG‐I (Goubau, Deddouche, & Reise Sousa, 2013; Kato et al., 2006). RIG‐1‐like receptors have an *N*‐terminal caspase activation and recruitment domain (CARD), which shows ligand‐dependent interaction with CARDs from other proteins, such as mitochondrial antiviral signalling protein (MAVS, Link to UniProt). MAVS activates IKK family kinases, such as TANK binding kinase (TBK1) and IKK‐ε, leading to the phosphorylation of IFN regulatory factors, such as IRF3 (Link to UniProt) and IRF7 (Link to UniProt). This induces the transcription of Type I IFN genes, such as IFN‐β and CCL5 (also known as RANTES) (Doyle et al., 2002; Fitzgerald et al., 2003; Sharma et al., 2003). MAVS present in peroxisomes is also able to recruit short‐acting, IFN‐independent defence factors (Dixit et al., 2010).

The ORF9b protein from SARS‐CoV has also been reported to target mitochondrial MAVS to limit the IFN response, as well as triggering proteolysis of dynamin‐like protein 1 (Link to UniProt) thereby prompting the formation of mitochondria‐associate autophagosomes claimed to create “havoc” in energy production in infected cells (Shi et al., 2014). In a very recent study, ORF9b of SARS‐CoV‐2 has been reported to interact with translocases of outer membrane 70 (Tom70, Link to UniProt) when expressed in HEK293 cells (Gordon, Jang, et al., 2020) Tom70 activates mitochondrial IRF3 (Liu, Wei, Shi, Shan, & Wang, 2010) and so this is a potential locus for pharmacological intervention, but as yet with no inhibitors described in the literature.

A number of other coronavirus proteins have been identified to influence the IRF3 pathway to restrict IFN production. This includes the MERS‐CoV PL_pro_ proteinase (Yang et al., 2014), as well as the ORF6 and nucleocapsid proteins from SARS‐CoV (Kopecky‐Bromberg, Martinez‐Sobrido, Frieman, Baric, & Palese, 2007). The ORF6 protein of SARS‐CoV has also been described to reduce the activity of a series of karyopherin‐dependent host transcription factors (Sims et al., 2013). Karyopherin is an importin, which traffics proteins between the cytoplasm and the nucleus (for review, see Kosyna & Depping, 2018; Guo, Fare, & Shorter, 2019).

Clearly, the induction and suppression of IFN production are central to numerous human diseases and have been extensively studied; the 'trick' to treat COVID‐19 will be to identify a novel angle for therapeutic exploitation.

### 6.2 Nsp1

Working with SARS‐CoV (not SARS‐CoV‐2), Pfefferle et al. (2011) used yeast two‐hybrid screens to identify interactions between the viral and human proteomes. They identified an interesting interaction between viral Nsp1 and a group of host peptidyl‐prolyl *cis‐trans*‐isomerases (PPIA, PPIG, PPIH and FKBP1A, FKBP1B), all of which modulate the calcineurin/NFAT pathway important in immune activation (reviewed by Hogan, Chen, Nardone, & Rao, 2003). The nsp1 protein acts on these to activate NFAT signalling and immune activation. Cyclosporine A, an inhibitor of this pathway, has been used for several decades to control transplant rejection and some autoimmune diseases and, in a simple in vitro assay, cyclosporine inhibited SARS‐CoV transcription/replication in (non‐immune‐system) cells (Pfefferle et al., 2011). SARS‐CoV‐2 has an nsp1 protein closely related to that of SARS‐CoV (Dong et al., 2020; Srinivasan et al., 2020), though its effect on the NFAT pathway seems not to have been reported. Nevertheless, cyclosporine has been shown to inhibit SARS‐CoV2 in an *in vitro* Vero cell‐based assay in a recent report (Jeon et al., 2020). It has therefore been suggested as a drug target (see, for example, Li & De Clercq, 2020). It may seem paradoxical to suggest an inhibitor of immune activation as a treatment for viral disease, but for the subgroup of patients that might suffer cytokine storms (Mehta et al., 2020), the double‐action might be useful.

### 6.3 *ORF3a*, *ORF6*, *ORF8* and other viral proteins

The ORF3a protein of SARS‐CoV appears to bind calcium in a cytoplasmic domain (Minakshi, Padhan, Rehman, Hassan, & Ahmad, 2014) and to elicit a response from the innate immune system by enhancing the ubiquitination of apoptosis‐associated speck‐like protein containing a CARD (Asc, Link to UniProt), which in turn activates the NLRP3 inflammasome and caspase 1 (Siu et al., 2019). The potential for targeting Asc and the NLRP3 inflammasome for therapeutic benefit in inflammatory conditions has recently been reviewed (Mangan et al., 2018), although there are no inhibitors in the clinic as yet.

In SARS‐CoV, the *Orf8a* and *Orf8b* genes became separated as the disease progressed by a 29‐nucleotide deletion (Chinese SARS Molecular Epidemiology Consortium, 2004; Oostra, de Haan, & Rottier, 2007). The *Orf8a* gene of SARS‐CoV encodes a short (31 aa, 1 TM, Link to UniProt) protein, which forms a cation channel of predicted pentameric structure (Chen et al., 2011). In SARS‐CoV‐2 and a bat‐derived coronavirus, in contrast to the SARS‐CoV‐2 genome, *Orf8* encodes a continuous 121 aa ORF8 protein (Cagliani, Forni, Clerici, & Sironi, 2020). Given that sequence analysis of different strains of SARS‐CoV‐2 suggests that the *Orf8* locus displays only limited evidence of positive selection (Cagliani et al., 2020), it seems germane to investigate the profile of ORF8 protein in more depth. Sequence comparisons led to prediction of secondary structure composed of an α‐helix and a β‐sheet containing six strands (Chan et al., 2020), but there appears not to be any literature as to whether this entity is a functional ion channel.

In a very recent report, the ORF14 protein (Link to UniProt) of SARS‐CoV‐2 has been reported to interact with NOD‐like receptor X1 (NLRX1), proteinase‐activated receptor 2 (PAR2/F2RL1), and NEDD4 family‐interacting protein 2 (NDFIP2, Link to UniProt), among other proteins of the IκB/NF‐κB pathway, when expressed in HEK293 cells (Gordon, Jang, et al., 2020). At the moment, there are no approved drugs targeting PAR2, although AZ3451 (Link to GtoP) acts as a negative allosteric modulator with pIC_50_ values of 5–23 nM (Cheng et al., 2017).

There is a limited insight into the roles or potential exploitability of the remaining range of other viral proteins (nsp2; nsp9; nsp11, proteins Orf3b; ORF6; ORF7a; ORF7b; ORF10).

### 7 ANIMAL MODELS OF SARS‐COV‐2 INFECTION

The spike glycoproteins in SARS‐CoV and MERS‐CoV are crucial for host specificity and jumping between species, for example, from bats to humans (Lu, Wang, & Gao, 2015) and from dromedary camels to humans (MERS‐CoV) and also the recent crossover of a HKU2‐related coronavirus to pigs as a swine acute diarrhoea syndrome (SADS‐CoV) (Zhou et al., 2018). SADS‐CoV appears to influence the innate immune system by reducing IFN‐β production evoked through IPS‐1 and RIG‐I pathways, but not through IRF3, TBK1 and IKKε (Zhou et al., 2020).

ACE2, as an anchoring point for the spike glycoprotein, is present throughout the animal kingdom, but small structural differences are critical for interaction with the spike protein (Li, Qiao, & Zhang, 2020; Luan, Lu, Jin, & Zhang, 2020). Key sequences of the spike protein from SARS‐CoV and SARS‐CoV‐2 are responsible for binding to ACE2. Luan et al. (2020) found that the key residues in S protein, from SARS‐CoV and SARS‐CoV‐2, recognized in ACE2 from dog, cat, pangolin and *Circetidae* mammals (simulated through homology modelling) were broadly similar. Mouse ACE2 is inefficient in prompting entry of both SARS‐CoV and SARS‐CoV‐2 (Fung et al., 2020). Cats and dogs suffer from their own specific coronavirus infections (e.g. canine respiratory coronavirus and feline coronavirus) without significant crossover to humans. A very recent report has suggested that cats and ferrets are sensitive to SARS‐CoV‐2, but dogs, pigs, chickens and ducks are much less sensitive (Shi, Wen, Zhong, Yang, Wang, Huang, Liu, He, Shuai, Sun, & Zhao, 2020). Ferrets, which have previously been used as models for respiratory tract infections, retained the SARS‐CoV‐2 virus in the respiratory tract, while the infection was transmitted between cats by aerosol (which may have implications for confinement); infected cats subsequently produced antibodies (Shi, Wen, et al., 2020).

The Syrian hamster has been used as a model for SARS‐CoV (de Wit et al., 2013; Roberts et al., 2005; Roberts et al., 2006) and studies with mice and Syrian hamsters are ongoing with SARS‐CoV‐2. In a small study where three juvenile (3–5 years old) and two mature (15 years old) rhesus macaques were infected intratracheally with the SARS‐CoV‐2 virus, all the monkeys showed symptoms of inflammation and interstitial pneumonia, with a greater apparent severity in the older animals (Yu et al., 2020).

Thus, while there is intensive research in animal models, a clearly validated model is still not apparent.

### 8 INTER‐INDIVIDUAL VARIATIONS IN SUSCEPTIBILITY

Given the similarities in the viruses and their symptoms, there is clearly a value to comparing the profiles of sufferers from the original SARS and subsequent MERS outbreaks with COVID‐19 to evaluate the risk factors associated with each event individually and collectively. A detailed consideration is beyond the scope of this review, but there are some obvious questions to ask (not in an order of priority).

1. What factor/s determine resistance to infection?
  It is apparent that many individuals who test positive for SARS‐CoV‐2 infection only experience 'mild'symptoms, others suffer a level of debilitation requiring hospitalization with limited supervision and a third group requires assisted breathing.
2. Is blood group a predictor?
  There is preliminary evidence (as yet, not peer‐reviewed) suggesting that people with type A blood might be more at risk of COVID‐19 than those with other blood types (Zhao et al., 2020).
3. Are there 'simple' genetic markers which predict this variation?
  For example, are single nucleotide polymorphisms/haplotypes for key targets (including ACE2, TMPRSS2, etc., Delanghe, Speeckaert, & De Buyzere, 2020) associated with higher or lower damage in humans infected with SARS‐CoV, MERS‐CoV, or SARS‐CoV‐2?
4. Reports suggest that there is a preponderance of male victims of COVID‐19, for example in Spain (Instituto de Salud Carlos III, Ministry of Science & Innovation, Spain. Retrievedon 2020‐03‐25, referring to data from 2020‐03‐24). What might be the cause of this sexual divergence?
5. Is smoking history a predictor of variation?
  One potential explanation for the relatively high proportion of male victims has been suggested to be previous smoking history (Cai, 2020; Olds & Kabbani, 2020; Vardavas & Nikitara, 2020), clearly a general risk factor for many diseases. Is there evidence from the SARS and MERS outbreaks to suggest a commonality of susceptibility?
6. What is the impact of contracting the virus on individuals with other underlying conditions?
  For example, what are the mechanism/s underlying why some sufferers of hypertension and/or diabetes might be at higher risk (https://www.immunopaedia.org.za/breaking-news/why-are-hypertension-and-diabetes-patients-at-high-risk-of-severe-covid-19/)? Is there evidence that patients on ACE inhibitors or angiotensin receptor antagonists were at higher risk with SARS‐CoV and MERS‐CoV infections and, currently, for SARS‐CoV‐2 infection?
7. How will the evolution of the virus alter rates of infection and the severity of symptoms?
  Some level of mutation is to be expected and indeed has been noted for the SARS‐CoV‐2. At the moment, it is too early to identify the significance of any influence of these mutations on the course of COVID‐19.

Some of these questions are more tractable since the SARS and MERS outbreaks because of the strides being made in sophisticated molecular biological techniques (e.g. NextGen Sequencing). An additional distinction compared to the previous outbreaks is the major increase in patient numbers associated with COVID‐19, allowing greater comparisons to be made in many more geographical locations.

Inevitably, other questions will form as greater detail becomes available.

## 9 CONCLUSION AND RECOMMENDATIONS

This review has concentrated on the prevailing hypothesis that an essential first step in infection is SARS‐CoV‐2 binding to ACE2 and for TMPRSS2 to prime the viral spike protein. We further hypothesize that both proteins must be expressed on a target cell for the virus to gain entry. TMPRSS2 has an extensive cellular expression profile, whereas ACE2 is more limited and is usually at low levels, unless increased by risk factors such as sex, age and smoking history, so is likely to be rate‐limiting. Other potential target proteins such as cathepsin L or B^0^AT1 may also prove important.

Currently, although there are no drugs approved for the treatment of patients with COVID‐19, the pandemic has triggered a stampede into clinical trials with both approved and investigational agents. The pharmacological rationale for these trials is sometimes obscure, but there is a logic to focus on viral entry and replication, as well as limiting the host immune response.

For the immediate term, the highest priority would be to investigate known antivirals to mitigate effects of COVID‐19. For the longer term, a vaccine (for review, see Amanat & Krammer, 2020) seems to hold the most promise to reduce COVID‐19 damage. There is also a role in the mid‐term, however, for drug discovery conducted in mainstream pharmacology labs. The goal here would be an international co‐ordinated approach to drug repurposing, examining the spectrum of licensed drugs (likely to be less than 2,000, varying dependent on jurisdictions). These would ideally be screened in a co‐ordinated, blinded fashion in multiple labs simultaneously to account for any minor methodological differences. This requires the reopening of screening and protein biosynthesis labs closed at the start of the pandemic, while ensuring that workers are kept safe.

If one were to write a Target Product Profile for a drug to treat COVID‐19, several parallel profiles can be identified. There are clear considerations, which may be identified as desirable pharmacodynamic, screening methodologies, drug metabolism and pharmacokinetic and formulation profiles.

From a pharmacodynamic perspective, a priority would be to screen the proteinases identified in this review (ACE2, TMPRSS2, ADAM17, cathepsin L, cathepsin B, PL_pro_ and 3CL_pro_). A second parallel stream would assess inhibitors of the viral RNA polymerase and endoribonuclease complexes, as well as the ion channel functions of the viral envelope (and potentially the Orf8 protein). Clearly, there are multiple other targets, which might bear fruit, and so further studies should assess the tractability of B^0^AT1/SLC6A19, B^0^AT3/SLC6A18, IMPDH2 and HAS2. Further, the molecular mechanism of action of ivermectin should be assessed, since it has recently been shown to inhibit in vitro SARS‐CoV‐2 replication (Caly, Druce, Catton, Jans, & Wagstaff, 2020). This agent is used clinically as an anthelmintic, probably through blocking invertebrate glutamate receptors although it also inhibits mammalian glycine receptors and acts as a positive allosteric modulator of other mammalian ligand‐gated ion channels.

From a screening aspect, biophysical and biochemical screens would probably take a matter of days to weeks. Following mass availability of the recombinant proteins involved, the capacity for inhibition should be assessed using a library of already approved drugs. Biophysical methods can be applied, such as surface plasmon resonance or biolayer interferometry, to monitor the affinity of interaction between host ACE2 and viral spike glycoprotein in the presence of these agents, as well as the relevant proteins where multimerization is critical, such as the trimeric spike glycoprotein. Assessing the remainder of the targets would likely adopt standard, fluorescent‐based pharmacological approaches.

If the assay involves the use of viral proteins, the constructs should acknowledge the inevitable mutations which the viral genome has/will undergo.

An overarching priority for the in vitro screening would be to recognize and replicate, as much as possible, relevant features of the virus and its life cycle. This would include post‐translational modifications of the viral proteins, such as glycosylation of the spike and membrane proteins. Additionally, while the high throughput screens described above for identifying inhibitors associated with components of the viral entry system, such as ACE2, should be confirmed in more translational assays, examples of which have been described for HIV cell entry in an automatable format (Bradley et al., 2004).

A desirable element would also be to minimize adverse effects on the cardiovascular and respiratory systems, given the high incidence of damage described associated with those systems (Esler & Esler, 2020; Li, Yang, et al., 2020; Lippi, Lavie, & Sanchis‐Gomar, 2020). Candidate drugs should also not increase activity of the IL‐6 (or any other pro‐inflammatory cytokine) pathway to avoid provoking a cytokine storm.

If a similar approach were taken to the ways in which targeted therapy is applied for certain types of cancer, there would be an increased benefit in a multimodal strategy. Thus, in cancers where EGF and EGF receptors are involved, it is possible to target the ligand using chelating antibodies, to antagonize the receptor using blocking antibodies, to use specific antibodies to prevent dimerization of the receptor and to inhibit the catalytic activity of the receptor with small molecular inhibitors. It should be possible to reproduce this approach by simultaneously targeting several steps in the viral cycle (while naturally being cognisant of the potential for phenomena of drug:drug interactions, for instance, in terms of convergent pathways of drug metabolism). This approach, enacted for the treatment of hepatitis C and human immunodeficiency viruses, for example, should also show benefit in reducing the capacity for drug‐driven mutation in the enzyme.

From a DMPK perspective, a beneficial profile for any agent would avoid drug:drug interactions by not converging on key metabolic enzymes and/or transporters. Ideally, a once‐daily treatment regimen would be optimal, but if more frequent administration were needed, there is likely to be good patient adherence, given the public response to 'spatial distancing'. From a formulation perspective, prophylactic use or for treatment of mild symptoms, an oral administered or inhaled formulation would be appropriate. For more severe cases, where breathing is significantly impaired, an inhaled aerosolized version maybe difficult to administer effectively; in this circumstance, a soluble version to be applied i.v. is likely to be useful.

Micro‐organisms, such as viruses and bacteria, continue to evolve to evade our immune systems and previous pandemics contributed to the decline and fall of civilizations. There is a widespread hope that the current pandemic will be controlled by the rapid development of a safe and efficacious vaccine. Clearly, there are major successes with vaccines targeting viral disease, but, to date, no vaccine has been successfully produced to protect against human betacoronaviruses such as those causing SARS and MERS. On the contrary, multiple viral diseases have been successfully controlled by pharmacological agents. HIV‐AIDS became more widespread in the last century and was associated with high morbidity and mortality. As a result of the discovery of novel pharmacological treatments, including specific antivirals, it is now a chronic condition, and a cure has been effected in at least two individuals. Similarly, the highly variable hepatitis C virus has resisted vaccines but can be treated with direct antiviral agents allowing elimination of the virus in a very high proportion of those treated. This gives us hope that the roadmap outlined in this review may provide some relief from COVID‐19 (and indeed for viral threats yet to come).

### 9.1 Nomenclature of targets and ligands

Sequencing analysis of the novel virus has identified a high level of similarity with the virus identified to cause the severe acute respiratory syndrome (SARS) outbreak in China in 2002/03/04, which was known as the SARS coronavirus or SARS‐CoV. Provisionally named as 2019‐nCoV, the virus has been renamed SARS‐CoV‐2 (Coronaviridae Study Group of the International Committee on Taxonomy of Viruses, 2020). For the purposes of this document, the virus is described as SARS‐CoV‐2, while the infectious disease is named as COVID‐19 (World Health Organization, 2020). One of the positive aspects of the emergence of SARS‐CoV‐2 and COVID‐19 is the rapidity at which aspects like genome sequencing (for example, Lu et al., 2020; Wu et al., 2020) and 3D structures (for example, Yan et al., 2020) have been described.

Protein targets and drugs in the current review follow nomenclature as presented on the GuidetoPHARMACOLOGY.org website (Alexander, Ball & Tsoleridis. SARS‐CoV‐2 proteins, accessed on 2020‐04‐24) and the Concise Guide to PHARMACOLOGY 2019/20 (Alexander et al., 2019).

## AUTHOR CONTRIBUTIONS

The document was conceived in discussions among S.P.H.A., J.A., J.D., E.F., S.D.H., F.L.S., A.J.P., C.S., and M.S.; it was initially drafted by S.P.H.A., and all the co‐authors contributed text and checked the manuscript; all the authors read and agree to submission of the manuscript.

## CONFLICT OF INTERESTS

The authors declare no conflicts of interest.
